# Assessing Resident Understanding of Common Pediatric Rashes on Skin of Color

**DOI:** 10.1089/heq.2023.0109

**Published:** 2023-09-04

**Authors:** Gurbaksh Esch, Brittany Lane, Christopher Bergsman

**Affiliations:** ^1^ Department of Pediatrics, Hurley Children's Hospital, Flint, Michigan, USA.; ^2^ Department of Pediatrics, Michigan State University, College of Human Medicine, Flint, Michigan, USA.; ^3^ Department of Pediatrics, Corewell Health, Grand Rapids, Michigan, USA.; ^4^ Department of Pediatrics, Oakland University William Beaumont Medical School, Rochester, Michigan, USA.

**Keywords:** skin of color, dermatology, pediatrics, residency

## Abstract

**Purpose:**

Pediatric dermatological training lacks in skin of color education and exposure, contributing to health inequities.

**Methods:**

We collected data from a survey assessing comfort of diagnosis on SOC before and after the intervention of a presentation.

**Results:**

This study demonstrates an increase in comfort of diagnosis after lecture intervention.

**Conclusion:**

This highlights the need for further education to allow for increased confidence and knowledge with diagnosis, as well as mastery. It also demonstrates the importance of exposure to SOC dermatology in medical and resident training to improve health equity.

## Introduction

As efforts increase to raise awareness regarding health disparities in skin of color (SOC) representation within medical education, little has been done to rectify this matter through curricular integration. Lack of regulation regarding educational exposure to skin color, especially Fitzpatrick Types IV–VI during graduate medical education, creates negative implications for patient safety and care.^1,2^ Current teaching still disproportionately depicts White skin, which can lead to negative implications for residency.^3,4^ With dermatology concerns accounting for a substantial percentage of primary care visits, it is imperative that residency training programs evaluate their curriculum to address disparities in training to increase accuracy in diagnosing and treating patients of color.

To date, there is no established standardized curriculum dedicated to ensuring that pediatric SOC correlates with Accreditation Council of Graduate Medical Education (ACGME) or American Academy of Pediatrics guidelines.^5^ Moreover, outside of textbooks dedicated to pediatric patients with Fitzpatrick skin types IV–VI, widely used resources and publications have limited number of SOC imaging.^6^ Recent studies indicate that although 91% of rashes are managed by primary care physicians without referral over the past 3 years, 28.4% of SOC articles related to pediatric dermatology.^7^ Pediatricians play a pivotal role in identifying dermatological conditions that can decrease a patient's quality of life or prevent adverse outcomes. As the U.S. population continues to diversify, it is imperative that it is required for clinicians to be educated and trained to treat conditions in SOC.^8^

## Methods

This multi-institution medical education study was done at the following institutions: Beaumont Health, Hurley Medical Center, and Sparrow Hospital. These three institutions are in Michigan, in Royal Oak, Flint, and Lansing, respectively. The project was approved by the pediatric program leaderships and the IRB, including data sharing agreements.

Inclusion criteria encompassed subjects that are pediatric or medicine-pediatric residents. Exclusion criteria consisted of persons in medical school, students, and attending physicians.

Presurvey and postsurvey were administered, using a 5-point Likert scale. The surveys assessed previous dermatological medical education, comfort with diagnosis and treatment, and clinical exposure to skin pathologies in SOC.

The lecture covered a variety of topics including basic skin layer review, overview of melanin biology, understanding of Fitzpatrick skin types, defining SOC, and common pediatric rashes on SOC with brief overviews and multiple images.

The primary outcome of this study was to understand the relationship between the intervention of the lecture on common rashes on SOC and comfort in diagnosis of rashes on SOC. We hypothesized that this small-scale intervention would at least somewhat improve comfort in diagnosis of common rashes on SOC.

Secondary outcomes included assessing previous education on SOC dermatology including knowledge regarding healing of SOC, the helpfulness of the lecture including within clinical context and understanding, and the level of interest in learning about SOC dermatological pathologies.

## Results

The total impact of this project included ∼10 attending physicians, 13 medical students, and 40 residents. Pediatric residency programs from Beaumont Hospital, Hurley Children's Hospital, and Sparrow Hospital participated in the training. The vast majority of residents received “not much” or “some” training in dermatology in residency. In addition, the majority of residents reported “some” educational history on rashes of SOC. Most residents reported seeing SOC patients in their clinical setting, inpatient and/or outpatient “a sufficient amount” or “a lot” on a regular basis. However, most felt only “somewhat” comfortable in diagnosis of common pediatric rashes on SOC.

**FIG. 1.**
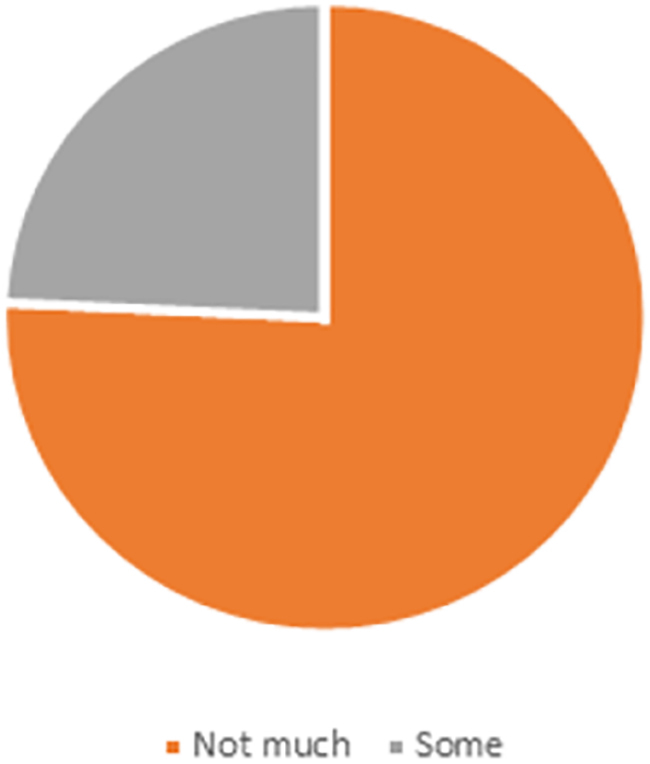
Dermatology experience in residency (lectures, exposure, and reading).

**FIG. 2.**
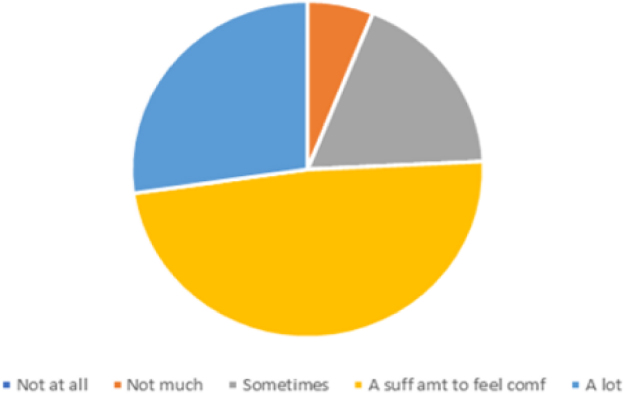
How often have you seen rashes on SOC in the clinical setting (inpatient/outpatient)?. SOC, skin of color.

The prelecture survey given to the pediatric residents revealed, similar to the literature, that the amount of dermatology experience/exposure during medical school and residency has been mainly “not much” or “some” (Fig. 1). With regard to SOC education, 100% reported that they have seen “not much” or “some” rashes on SOC during lectures, with 89% reporting “not much” or “some” when asked whether they have seen comparisons of SOC and white skin with regard to rashes. Though the education regarding SOC dermatology has been minimal, 88% reported that they see either “a sufficient amount,” “some,” or “a lot” of SOC rashes in their clinical settings, inpatient and outpatient (Fig. 2).

The comfort level regarding diagnosis of common pediatric rashes on SOC showed that 77% felt “somewhat” or “not very” comfortable. In addition to this gap of knowledge, there is also a lack of knowledge and comfort regarding treatment, with 89% reporting only “some” or “not much” education on treatment of rashes on SOC. Finally, when asked whether they have been provided educational materials regarding rashes on SOC, 78% reported “no,” though 100% of participants reported their interest in learning about rashes on SOC.

The postlecture surveys data showed an increase in knowledge and comfort. There was a 49% increase in those who felt “fairly” comfortable diagnosis pediatric rashes on SOC and the rest (29%) reported they felt “very” comfortable. As already stated, a majority of the residents reported at least some exposure to SOC rashes in their clinical setting, reporting that this lecture was “very helpful” (57%) or “fairly helpful” (43%). When specifically discussing healing on SOC, 71% felt it was “very helpful” in explaining this. Finally, 57% reported that they were “very likely” to recommend this lecture to peers.

After the intervention, using a paired *t*-test, we note a statistically significant change in comfort in diagnosing SOC rashes. We can see that the mean of comfort increased by ∼1 U on the Likert scale (Table 1).

**Table 1.** Paired *T*-Test Pre- and Postcomfort in Diagnosis of Skin of Color Rashes

	Mean		***N***	Standard deviation		Standard error mean		
Pretest comfort diagnosing SOC rashes	2.5926		27	0.63605		.12241		
Post-test comfort diagnosing SOC rashes	3.6296		27	0.74152		0.14271		

	Mean	Standard deviation	Standard error mean	95% Confidence interval of the difference		***T***	Df	Significance (two-tailed)
				**Lower**	**Upper**			
Pretest comfort diagnosis SOC rashes—Post-test comfort diagnosing SOC rashes	−1.03704	0.64935	0.12497	−0.12391	–0.78016	−8.298	26	<0.001

SOC, skin of color.

Comments on improvement included the want for “a wider variety of skin color shades for the rashes,” a request for “more rare rashes on skin of color,” and “more of a description and explanation for each rash.”

## Conclusion

The ACGME requires that residents learn the process of quality improvement and health disparities and utilize that knowledge to improve equity. This multi-institutional pilot project examined the current state of medical education surrounding SOC dermatology education.

Limitations include the sample size. Pursuing a multi-institutional project allowed for greater numbers and more ability to generalize; however, there is significant room for improvement and more recruitment. In addition, though this single intervention yielded positive results with regard to comfort with diagnosis, mastery is only achieved through longitudinal and continued intervention.

## Discussion

Recommendations for the future of health equity in dermatology education have been reported in other studies, such as increase in SOC representation in medical journals and textbooks, increased research in SOC dermatology diagnosis and management, and increased exposure to SOC images during educational lectures and sessions.^1,9,10^ Incorporating SOC images into medical school and residency lectures is a simple and feasible way to increase exposure and comfort with diagnosis, as evidenced by this project.

Beyond the scope of this project and the above recommendations, creating policies at the level of accreditation of medical schools and residency programs that increase exposure to SOC images and education in recognizing health disparities in the contexts of social determinants of health and racial/historical trauma can create long-term sustainable changes toward health equity.

## Abbreviations Used

ACGME: Accreditation Council for Graduate Medical Education
SOC: skin of color
